# In vitro antibacterial and antibiotic-potentiation activities of the methanol extracts from *Beilschmiedia acuta, Clausena anisata*, *Newbouldia laevis* and *Polyscias fulva* against multidrug-resistant Gram-negative bacteria

**DOI:** 10.1186/s12906-015-0944-5

**Published:** 2015-11-22

**Authors:** Simplice B. Tankeo, Pierre Tane, Victor Kuete

**Affiliations:** Department of Biochemistry, Faculty of Science, University of Dschang, P.O. Box 67, Dschang, Cameroon; Laboratory of Natural Products Chemistry, Department of Chemistry, Faculty of Science, University of Dschang, Dschang, Cameroon

**Keywords:** Antibacterial activities, *Beilschmedia acuta*, Gram-negative bacteria, Multi-drug resistance, Lauraceae

## Abstract

**Background:**

The present study was designed to investigate the antibacterial activities of the methanol extracts from different parts of *Beilschmedia acuta* Kosterm (Lauraceae)*, Clausena anisata* (Willd) Hook (Rutaceae), *Newbouldia laevis* Seem (Bignoniaceae) and *Polyscias fulva* (Hiern) Harms (Araliaceae) as well as their synergistic effects with antibiotics against a panel of Gram-negative bacteria, including multi-drug resistant (MDR) phenotypes expressing active efflux pumps.

**Methods:**

Broth microdilution method was used to determine the minimum inhibitory concentrations (MICs) and the minimum bactericidal concentrations (MBCs) of the extracts, as well as those of antibiotics in association with the most active ones, *B. acuta, N. laevis* and *P. fulva*.

**Results:**

MIC values obtained indicate that extracts from the bark of *B. acuta* were active on all the 26 tested Gram-negative bacteria, with MICs ranging from values below 8 to 256 μg/mL. Other samples displayed selective activities, their inhibitory effects being observed on 9 (34.62 %) of the 26 bacterial strains for *N. laevis* leaves extract, 6 (23.10 %) for both *C. anisata* leaves and roots extracts, 7 (26.9 %) and 4 (15.4 %) for leaves and roots extracts of *P. fulva* respectively. Extract from *B. actua* bark displayed the best antibacterial activity with MIC values below 100 μg/mL against 16 (61.5 %) of the 26 tested microorganisms. The lowest MIC values (below 8 μg/mL) were obtained with this extract against *Escherichia coli* W3110 and *Klebsiella pneumoniae* ATCC11296. The MIC values of this extract were lower than those of ciprofloxacin against *E. coli* W3110, *Enterobacter aerogenes* ATCC13048, CM64 and *Providencia stuartii* NAE16. At MIC/2, the best percentages of synergistic effects (100 %), were obtained with *B. acuta* bark extract and tetracycline (TET) as well as with *P. fulva* leaves extract and TET and kanamycin (KAN).

**Conclusion:**

The overall results of the present study provide information for the possible use of the studied plants and mostly *Beilschmedia acuta* in the control of bacterial infections including MDR phenotypes.

**Electronic supplementary material:**

The online version of this article (doi:10.1186/s12906-015-0944-5) contains supplementary material, which is available to authorized users.

## Background

Fighting multi-drug resistant (MDR) Gram-negative (MDRGN) bacteria remains a challenging issue worldwide. Microbial infections involving MDRGN bacteria constitute a major public health problem in developing countries [1] where the high cost of antibiotics makes them unaffordable to the majority of the population. Clinically, the continuous emergence of MDRGN bacteria drastically reduced the efficacy of antibiotic arsenal and, consequently, increased the frequency of therapeutic failure [2]. Therefore, the discovery of new antimicrobial agents is still relevant nowadays. Also, the shortcomings of drugs available today and scarcity of novel antibiotics propel the discovery of new chemotherapeutic agents from medicinal plants [3]. Approximately 60 % of the world population still relies on medicinal plants for their primary healthcare [4]. Medicinal plants have been used as a source of remedies since ancient times in Africa. In addition, promising new concepts such as the efflux pump inhibitors [5, 6], and synergy between antibiotics and phytochemicals are now being developed. The ability of several African medicinal plants to inhibit the growth of MDRGN bacteria, as well as their ability to potentiate the activity of commonly used antibiotics was previously reported. Some of these plants include *Dorstenia psilurus*, *Dichrostachys glomerata* and *Beilschmiedia cinnamomea* [7–9].

In our continuous search of plant extracts with antibiotic-potentiating activity to combat MDR bacteria, the present work was designed to investigate the antibacterial activity of four Cameroonian medicinal plants used traditionally in the treatment of bacterial infections, namely *Beilschmiedia acuta* Kosterm (Lauraceae), *Clausena anisata* (Willd) Hook (Rutaceae), *Newbouldia laevis* Seem (Bignoniaceae) and *Polyscias fulva* (Hiern) Harms (Araliaceae), against MDRGN expressing active efflux *via* the Resistance-Nodulation Cell Division (RND)-type pumps. In the treatment of infectious diseases, *Beilschmiedia acuta* is traditionally used for gastrointestinal infections [10], *Clausena anisata* for fungal, bacterial and viral infections, *Newbouldia laevis* for bacterial and fungal infections [11–14], dysentery, worms, malaria, dental caries and diarrhea [15] and *Polyscias fulva* for venereal infections [16, 17].

## Methods

### Plant material and extraction

All medicinal plants used in the present work were collected in different areas of Cameroon between January and April 2012. The plants were identified at the National Herbarium (Yaounde, Cameroon), where voucher specimens were deposited under the reference numbers (Table 1). Air-dried and powdered plant material was weighed (300 g) and soaked in 1 L of methanol (MeOH) for 48 h at room temperature. The filtrate obtained through Whatman filter paper No.1 was concentrated under reduced pressure in a vacuum to obtain the crude extract. All crude extracts were kept at 4 °C until further use.

**Table 1:** Information of plants used in this study

Plants samples (family) and Herbarium Voucher number^a^	Part used and extraction yield (%)^b^	Area of plant collection (Geographic Coordinates*)*	Traditional treatment	Bioactive (or potentially active) compounds isolated from plants	Biological activities of crude extract^c^
*Beilschmiedia acuta* Kosterm (Lauraceae) 37335/HNC	Leaves (18.40 %), fruits (20.22 %) and barks (36.46 %)	Lebialem, South-West Region of Cameroon; (4°10′N 9°14′E/4.167°N 9.233°E)	Cancer and gastrointestinal infections [10].	Flavonoids, triterpenes, phenols, saponins, alkaloids [10].	Cytotoxicity towards leukemia, breast, glioblastoma, colon and liver cancer cell lines [10].
*Clausena anisata* (Willd) Hook (Rutaceae) 44242/ HNC	Leaves (16.31 %) and roots (13.%)	Lebialem, South-West region of Cameroon	Diabetes, anti-hypertensive, anti-nociceptive, malaria, fungal, bacterial and viral infections, inflammation, heart and mental disorders, constipation, convulsions, impotence and sterility [43–46]	Essential oils (sabinene, β-pinene, pulegone, 1,8 -cineole, estragole, [42]; carbazole alkaloids, coumarins, limonoids [46, 47].	Antimicrobial: Essential oil active against *Sa, Sp, Esp*, *St, Pa* [41, 42]
*Newbouldia laevis* Seem. (Bignoniaceae) 29469/HNC	Leaves (18.75 %), and barks (19.35 %)	Melon, Littoral region of Cameroon (04°33'53"N 09°38'04"E)	Cancers, spasms, infectious diseases, male infertility and diabetes [11, 12], coagulant or anti-hemorrhagic properties; digestive threats, urogenital and pulmonary infections[13, 14]; Dysentery, worms, malaria, sexually transmitted diseases, dental caries and diarrhea [15].	Tannins, triterpenoids, mucilages and reducing compounds, flavonoids, steroids, alkaloids, cardiac glycosides [10, 14, 48].	Antimicrobial: active against *Ca, Ck, Sa, Sf, Ec, Pa, Sp, Pv, Kp, St, Sd, Ng Mtb, Ms* [14, 39, 49].
*Polyscias fulva* (Hiern) Harms. (Araliaceae) 60407/HNC	Leaves (15.62 %), roots (17.56 %) and barks (19.01 %)	Dschang, West region of Cameroon (6°30′N 10°30′E/6.500°N 10.500°E)	Malaria, fever, mental illness [50]; venereal infections and obesity [16, 17] and cancer [10]	Polysciasoside A, kalopanax-saponin B, alpha-hederin [51, 52]	Inhibition of microsomal lipid peroxidation [53]

^a^Plants were identified at the Cameroon National Herbarium (HNC); ICNA: Voucher with no identification code at the HNC; ^b^The percentage of the methanol extract; ^c^Microorganisms[*Bs Bacillus subtilis*, *Ca Candida albicans*, *Ck Candida krusei*, *Mm Mucor miehei*, *Cv Chlorella vulgaris*, *Cs Chlorella sorokiniana*, *Ec Escherichia coli*, *Esp Enterococcus species*, *Mtb Mycobacterium tuberculosis*, *Ms Mycobacterium smegmatis*, *Ng Neisseria gonorrhoeae*, *Pa Pseudomonas aeruginosa*, *Sf Streptococcus faecalis*, *Pv Proteus vulgaris*, *Sa Staphylococcus aureus*, *Sp Streptococcus pneumoniae*, *St Salmonella typhimurium*, *Kp Klepsiella pneumoniae*, *Sd Shigella dysenteriae*, *Ss Scenedesmus subspicatus*, *Sv Streptomyces viridochromogene*u]

### Antimicrobial assays

#### Chemicals for antimicrobial assays

Tetracycline (TET), ciprofloxacine (CIP), chloramphenicol (CHL), ampicillin (AMP) and kanamycin (KAN) (Sigma-Aldrich, St Quentin Fallavier, France) were used as reference antibiotics (RA). *p*-Iodonitrotetrazolium chloride (INT, Sigma-Aldrich) was used as a microbial growth indicator [18, 19].

### Microbial strains and culture media

The studied microorganisms included sensitive and resistant strains of *Pseudomonas aeruginosa, Klebsiella pneumoniae, Enterobacter aerogenes, Escherichia coli* obtained from the American Type Culture Collection (ATCC). Their bacterial features are summarized in Table 2. Nutrient agar was used to activate the tested Gram-negative bacteria [20].

**Table 2:** Bacterial strains used and their features

Strains	Features and References	
*Escherichia coli*		
ATCC10536	Reference strain	
AG100	Wild-type *E. coli* K-12	[54]
AG100A	AG100 *ΔacrAB*::KAN^R^	[34, 54, 55]
AG100A_TET_	Δ*acrAB* mutant AG100, with over-expressing *acrF* gene; TET^R^	[54]
AG102	Δ*acrAB* mutant AG100, owing *acrF* gene markedly over-expressed; TET^R^	[56, 57]
MC4100	Wild type *E. coli*	[58]
W3110	Wild type *E. coli*	[58, 59]
*Enterobacter aerogenes*		
ATCC13048	Reference strains	
CM64	CHL^R^ resistant variant obtained from ATCC13048 over-expressing the AcrAB pump	[60]
EA3	Clinical MDR isolate; CHL^R^, NOR^R^, OFX^R^, SPX^R^, MOX^R^, CFT^R^, ATM^R^, FEP^R^	[61, 62]
EA27	Clinical MDR isolate exhibiting energy-dependent norfloxacin and chloramphenicol efflux with KAN^R^ AMP^R^ NAL^R^ STR^R^ TET^R^	[61, 62]
EA289	KAN sensitive derivative of EA27	[63]
EA294	EA289 a*crA::*KAN^R^	[63]
EA298	EA 289 *tolC::*KAN^R^	[63]
*Enterobacter cloacae*		
ECCI69	Clinical MDR isolates, CHL^R^	[7]
BM67	Clinical MDR isolates, CHL^R^	[7]
*Klebsiella pneumoniae*		
ATCC12296	Reference strains	
KP55	Clinical MDR isolate, TET^R^, AMP^R^, ATM^R^, CEF^R^	[64]
KP63	Clinical MDR isolate, TET^R,^ CHL^R^, AMP^R,^ ATM^R^	[64]
K24	AcrAB-TolC, Laboratory collection of UNR-MD1, University of Marseille, France	[7]
K2	AcrAB-TolC, Laboratory collection of UNR-MD1, University of Marseille, France	[7]
*Providencia stuartii*		[65]
NEA16	Clinical MDR isolate, AcrAB-TolC	
ATCC29916	Clinical MDR isolate, AcrAB-TolC	
PS2636	Clinical MDR isolate, AcrAB-TolC	
PS299645	Clinical MDR isolate, AcrAB-TolC	
*Pseudemonas aeruginosa*		
PA 01	Reference strains	
PA 124	MDR clinical isolate	[66]

^a^AMP, ATM^R^, CEF^R^, CFT^R^, CHL^R^, FEP^R^, KAN^R^, MOX^R^, STR^R^, TET^R^. Resistance to ampicillin, aztreonam, cephalothin, cefadroxil, chloramphenicol, cefepime, kanamycin, moxalactam, streptomycin, and tetracycline; *MDR* Multidrug resistant

### INT colorimetric assay for MIC and MBC determinations

The MIC determination on the tested bacteria was conducted using rapid *p*-iodonitrotetrazolium chloride (INT) colorimetric assay according to described methods [18] with some modifications [21, 22]. The test samples and RA were first dissolved in DMSO/Mueller Hinton Broth (MHB). The final concentration of DMSO was lower than 2.5 % and does not affect the microbial growth [23, 24]. The solution obtained was then added to Mueller Hinton Broth, and serially diluted two fold (in a 96- wells microplate). One hundred microlitre (100 μL) of inoculum 1.5 x 10^6^ CFU/mL prepared in appropriate broth was then added [21, 22]. The plates were covered with a sterile plate sealer, then agitated to mix the contents of the wells using a plate shaker and incubated at 37 °C for 18 h. The assay was repeated thrice. Wells containing adequate broth, 100 μL of inoculum and DMSO to a final concentration of 2.5 % served as negative control. The MIC of samples was detected after 18 h incubation at 37 °C, following addition (40 μL) of 0.2 mg/mL of INT and incubation at 37 °C for 30 min. Viable bacteria reduced the yellow dye to pink. The MIC was defined as the sample concentration that prevented the color change of the medium and exhibited complete inhibition of microbial growth [18]. The MBC was determined by adding 50 μL aliquots of the preparations, which did not show any growth after incubation during MIC assays, to 150 μL of adequate broth. These preparations were incubated at 37 °C for 48 h. The MBC was regarded as the lowest concentration of extracts, which did not produce a color change after addition of INT as mentioned above [21, 22].

Samples were tested alone and the best four extracts (those from the leaves and bark of *Beilschmedia acuta,* and from the leaves of *Newbouldia laevis* and *Polyscias fulva*) were also selected and tested in association with antibiotics at the sub-inhibitory concentrations (MIC/2 and MIC/5) [7–9] against nine MDR bacteria. Fractional inhibitory concentration (FIC) was calculated as the ratio of MIC_Antibiotic in combination_/MIC_Antibiotic alone_ and the results were discussed as follows: synergy (*≤*0.5), indifferent (0.5 to 4), or antagonism (*>*4) [25, 26]. All assays were performed in triplicate.

## Results

The antibacterial activities of methanol extracts from various parts of *Beilschmedia acuta, Clausena anisata, Newbouldia laevis* and *Polyscias fulva* are summarized in Table 3 (MIC values up to 1024 μg/mL are provided as supporting information; Additional file 1: Table S1). It can be observed that extracts from the bark of *B. acuta* were active on all 26 tested Gram-negative bacteria, with MICs ranging from values below 8 to 256 μg/mL. Other samples displayed selective activities, their inhibitory effects being observed against nine (34.62 %) of the 26 bacterial strains for *N. laevis* leaves extract, six (23.10 %) for both *C. anisata* leaves and roots extracts, seven (26.9 %) and four (15.4 %) for leaves and roots extracts of *P. fulva* respectively. Extract from the bark of *B. actua* showed the best antibacterial activity with MIC values below 100 μg/mL against 16/26 (61.5 %) of the tested microorganisms. The lowest MIC values below 8 μg/mL were obtained with this extract against *Escherichia coli* W3110 and *Klebsiella pneumoniae* ATCC11296. MIC values of this extract were lower than those of ciprofloxacin against *E. coli* W3110, *Enterobacter aerogenes* ATCC13048 and CM64 and *Providencia stuartii* NAE16 (Table 3). The bactericidal activities of studied samples were mostly noted with the extract from *B. acuta,* with MBC values observed against 23/26 (88.5 %) tested bacteria (see Additional file 1: Table S2, supporting information).

**Table 3:** MICs (μg/mL) of the crude extracts and ciprofloxacin on the panel of tested bacteria

Bacterial strains	Studied samples and MIC (μg/mL)										
	*Beilschmedia acuta*			*Clausena anisata*		*Newbouldia laevis*		*Polyscias fulva*			Reference drug
	L	B	F	L	R	L	B	L	B	R	CIP
*Escherichia coli*											
ATCC 10536	>256	64	>256	256	256	128	>256	>256	>256	>256	1
AG 100A	>256	128	>256	>256	>256	256	>256	>256	>256	>256	<0.5
AG 100	>256	16	>256	>256	>256	>256	>256	>256	>256	>256	16
AG 100A_Tet_	>256	256	>256	>256	>256	>256	>256	>256	>256	>256	64
AG 102	>256	64	>256	>256	>256	>256	>256	>256	>256	>256	4
MC 4100	256	128	256	256	256	128	>256	256	>256	256	16
W 3110	256	<8	256	256	256	128	>256	128	>256	128	32
*Enterobacter aerogenes*											
ATCC 13048	>256	16	>256	>256	>256	>256	>256	256	>256	>256	32
CM 64	>256	16	>256	>256	>256	>256	>256	>256	>256	>256	64
EA3	>256	64	>256	>256	>256	>256	>256	>256	>256	>256	16
EA27	>256	64	>256	>256	>256	128	>256	256	>256	>256	4
EA 294	>256	64	>256	>256	256	256	>256	>256	>256	>256	2
EA 289	>256	256	256	>256	>256	>256	>256	>256	>256	>256	128
EA 298	>256	256	256	>256	>256	>256	>256	>256	>256	>256	16
*Klebsiella pneumoniae*											
ATCC11296	128	<8	256	256	256	128	>256	128	256	128	<0.5
K2	>256	256	>256	>256	>256	>256	>256	>256	>256	>256	16
KP55	>256	32	>256	>256	>256	>256	>256	>256	>256	>256	4
KP63	128	64	128	256	>256	256	>256	128	>256	128	4
*Providencia stuartii*											
ATCC29916	>256	128	>256	>256	>256	>256	>256	>256	>256	>256	32
PS2636	256	64	256	256	128	128	>256	256	>256	>256	64
PS299645	>256	256	>256	>256	>256	>256	>256	>256	>256	>256	32
NAE16	>256	32	>256	>256	>256	>256	>256	>256	>256	>256	128
*Enterobacter cloacae*											
ECCI69	>256	256	>256	>256	>256	>256	>256	>256	>256	>256	256
BM67	>256	256	>256	>256	>256	>256	>256	>256	>256	>256	32
*Pseudomonas aeruginosa*											
PA01	>256	64	256	>256	>256	>256	>256	>256	>256	>256	16
PA124	>256	32	>256	>256	>256	>256	>256	>256	>256	>256	32

The tested extracts were obtianed from the leaves (L), bark (B), roots (R) or fruits (F); CIP: ciprofloxacin]; MIC and MBC data with values up to 1024 μg/mL are provided as supporting information (Additional file 1: Table S1)

Five commonly used antibiotics (CIP, TET, KAN, AMP and CHL) were combined with extracts from *B. acuta* leaves and bark and those from the leaves of *N. laevis* and *P. fulva* at their MIC/2 and MIC/5, as obtained on each of nine tested bacterial strains (Tables 4 and 5). Synergistic effects were observed with all tested extracts and all studied antibiotics on at least one of the nine selected bacteria. The best percentages of synergistic effect (100 %) were obtained at MIC/2 with *B. acuta* bark extract in combination with TET (Table 5) as well as with *P. fulva* leaves extract in association with TET and KAN (Table 5).

**Table 4:** MIC of antibiotics after the association of the extract of *Beilschmedia acuta* at MIC/2 and MIC/5 against selected MDR bacteria

Antibiotics^a^	Extract and concentration		Bacterial strains^b^, MIC (μg/mL) of antibiotics in the absence and presence of the extract and FIC in parenthesis									PBSS (%)
			AG102	AG100ATET	EA27	CM64	KP55	NAE16	BM67	PA01	PA124	
CIP		0	4	64	4	64	4	128	32	16	32	
	L	MIC/2	4 (1)^I^	64 (1)^I^	1 (0.25)^S^	64 (1)^I^	8 (2)^A^	128 (1)^I^	16 (0.50)^S^	16 (1)^I^	16(0.50)^S^	3/9 (27.27 %)
		MIC/5	4 (1)^I^	64 (1)^I^	2 (0.50)^S^	64 (1)^I^	8 (2)^A^	128 (1)^I^	32 (1)^I^	16 (1)^I^	32(1)^I^	1/9 (11.11 %)
	B	MIC/2	4 (1)^I^	32 (0.50)^S^	1 (0.25)^S^	64 (1)^I^	4 (1)^I^	128 (1)^I^	16 (0.50)^S^	16 (1)^I^	32(1)^I^	3/9 (27.27 %)
		MIC/5	4 (1)^I^	64 (1)^I^	2 (0.50)^S^	64 (1)^I^	4 (1)^I^	128 (1)^I^	32 (1)^I^	16 (1)^I^	32(1)^I^	3/9 (27.27 %)
TET		0	8	64	64	32	2	64	32	64	16	
	L	MIC/2	2 (0.25)^S^	32 (0.50)^S^	32 (0.50)^S^	8 (0.25)^S^	2 (1)^I^	32 (0.50)^S^	32 (1)^I^	16 (0.25)^S^	8(0.50)^S^	7/9 (77.78 %)
		MIC/5	4 (0.50)^S^	64 (1)^I^	64 (1)^I^	16 (0.50)^S^	2 (1)^I^	64 (1)^I^	32 (1)^I^	64 (1)^I^	8(0.50)^S^	3/9 (27.27 %)
	B	MIC/2	4 (0.50)^S^	32 (0.50)^S^	16 (0.25)^S^	16 (0.50)^S^	1 (0.50)^S^	16 (0.25)^S^	16 (0.50)^S^	32 (0.50)^S^	8(0.50)^S^	9/9 (100 %)
		MIC/5	4 (0.50)^S^	64 (1)^I^	64 (1)^I^	16 (0.50)^S^	1 (0.50)^S^	32 (0.50)^S^	32 (1)^I^	64 (1)^I^	16(1)^I^	4/9 (36.36 %)
KAN		0	-	16	128	4	16	16	64	4	128	
	L	MIC/2	8(˂0.03)^S^	4 (0.25)^S^	64 (0.50)^S^	2 (0.50)^S^	16 (1)^I^	16 (1)^I^	32 (0.50)^S^	4 (1)^I^	64(0.50)^S^	6/9 (66.67 %)
		MIC/5	64 (˂0.25)^S^	16 (1)^I^	128 (1)	4 (1)^I^	16 (1)^I^	16 (1)^I^	64 (1)^I^	2 (0.50)^S^	64(0.50)^S^	3/9 (27.27 %)
	B	MIC/2	8 (˂0.03)^S^	˂1 (˂0.06)^S^	32 (0.25)^S^	˂1 (˂0.25)^S^	<1 (<0.06)^S^	8 (0.50)^S^	16 (0.25)^S^	4 (1)^I^	32(0.25)^S^	8/9 (88.89 %)
		MIC/5	128 (˂0.50)^S^	8 (0.50)^S^	128 (1)^I^	2 (0.50)^S^	4 (0.25)^S^	16 (1)^I^	32 (0.50)^S^	<1 (<0.25)^S^	64(0.50)^S^	6/9 (66.67 %)
AMP		0	-	-	-	-	-	-	-	-	-	
	L	MIC/2	128 (˂0.50)^S^	128 (˂1)^S^	-(≥1)	-(≥1)	-(≥1)	- (≥1)	-(≥1)	-(≥1)	-(≥1)	2/9 (22.22 %)
		MIC/5	256 (˂1)^S^	-(≥1)	-(≥1)	-(≥1)	-(≥1)	- (≥1)	-(≥1)	128 (<0.50)^S^	-(≥1)	2/9 (22.22 %)
	B	MIC/2	128 (˂0.50)^S^	-(≥1)	256 (˂1)^S^	-(≥1)	256 (<1)^S^	256 (<1)^S^	-(≥1)	-(≥1)	256(<1)^S^	5/9 (55.55 %)
		MIC/5	256 (˂1)^S^	-(≥1)	-(≥1)	-(≥1)	-(≥1)	- (≥1)	-(≥1)	256 (˂1)^S^	-(>1)	2/9 (22.22 %)
CHL		0	32	64	64	-	8	64	128	16	128	
	L	MIC/2	16 (0.50)^S^	32 (0.50)^S^	64 (1)^I^	128 (˂0.50)^S^	16 (2)^A^	8 (0.13)^S^	64 (0.50)^S^	2 (0.13)	64(0.50)^S^	6/9 (66.67 %)
		MIC/5	32 (1)^I^	64 (1)^I^	64 (1)^I^	-(˃1)	16 (2)^A^	16 (0.25)^S^	64 (0.50)^S^	8 (0.50)^S^	128(1)^I^	3/9 (27.27 %)
	B	MIC/2	32 (1)^I^	32 (0.50)^S^	32 (0.50)^S^	256 (˂1)^S^	2 (0.25)^S^	16 (0.25)^S^	64 (0.50)^S^	4 (0.25)^S^	64(0.50)^S^	8/9 (88.89 %)
		MIC/5	32 (1)^I^	32 (0.50)^S^	64 (1)^I^	-(˃1)	2 (0.25)^S^	32 (0.50)^S^	128 (1)^I^	8 (0.50)^S^	64(0.50)^S^	5/9 (55.55 %)

^a^Antibotics [*TET* tetracycline, *CIP* ciprofloxacin, *KAN* kanamycin, *CHL* chloramphenicol, *AMP* ampicillin]. ^b^Bacterial strains: *Escherichia coli* [AG102, AG100Atet], *Pseudomonas aeruginosa* [PA01, PA124], *Enterobacter aerogenes* [CM64, EA27], *Enterobacter cloacae* [BM67], *Klebsiella pneumoniae* [KP55], *Providencia stuartii* [NAE16]. ^c^PBSS: percentage of bacteria strain on which synergism has been observed

(): fold increase in MIC values of the antibiotics after association with plants extract; *S* synergy, *I* indifference, *na* not applicable, *B* bart extract, *L* leaves extract, *FIC* fractional inhibitory concentration, (−): >256 μg/mL; 0: no extract (only antibiotic tested)

**Table 5:** MIC of antibiotics after the association of the extract of *Newbouldia laevis* and *Polysicas fulva* at MIC/2 and MIC/5 against selected MDR bacteria

Antibiotics^a^	Extract and concentration	Bacterial strains^b^, MIC (μg/mL) of antibiotics in the absence and presence of the extract and FIC in parenthesis									PBSS (%)
		AG102	AG100ATET	EA27	CM64	KP55	NAE16	BM67	PA01	PA124	
	*Newbouldia laevis*										
CIP	0	4	64	4	64	4	128	32	16	32	
	MIC/2	4 (1)^I^	64 (1)^I^	2 (0.50)^S^	32 (0.50)^S^	4 (1)^I^	128 (1)^I^	16 (0.50)^S^	16 (1)^I^	32(1)^I^	3/9 (27.27 %)
	MIC/5	4 (1)^I^	64 (1)^I^	2 (0.50)^S^	64 (1)^I^	4 (1)^I^	128 (1)^I^	32 (1)^I^	16 (1)^I^	32(1)^I^	1/9 (11.11 %)
TET	0	8	64	64	32	2	64	32	64	16	
	MIC/2	4 (0.50)^S^	16 (0.25)^S^	32 (0.50)^S^	4 (0.13)^S^	2 (1)^I^	32 (0.50)^S^	16 (0.50)^S^	32 (0.50)^S^	8(0.50)^S^	8/9 (88.89 %)
	MIC/5	8 (1)^I^	32 (0.50)^S^	64 (1)^I^	8 (0.25)^S^	0.50 (0.25)^S^	64 (1)^I^	32 (1)^I^	64 (1)^I^	8(0.50)^S^	4/9 (36.36 %)
KAN	0	-	16	128	4	16	16	64	4	128	
	MIC/2	4 (˂0.03)^S^	8 (0.50)^S^	64 (0.50)^S^	2 (0.50)^S^	16 (1)^I^	8 (0.50)^S^	32 (0.50)^S^	2 (0.50)^S^	32(0.25)^S^	8/9 (88.89 %)
	MIC/5	64 (˂0.50)^S^	16 (1)^I^	128 (1)^I^	4 (1)^I^	1 (0.06)^S^	8 (0.50)^S^	32 (0.50)^S^	4 (1)^I^	64(0.50)^S^	5/9 (55.55 %)
AMP	0	-	-	-	-	-	-	-	-	-	
	MIC/2	-(≥1)	-(≥1)	256 (˂1)^S^	-(≥1)	-(≥1)	256 (˂1)^S^	-(≥1)	-(≥1)	-(>1)	2/9 (22.22 %)
	MIC/5	-(≥1)	-(≥1)	-(≥1)	-(≥1)	256 (˂1)^S^	- (≥1)	-(≥1)	-(≥1)	-(>1)	1/9 (11.11 %)
CHL	0	32	64	64	-	8	64	128	16	128	
	MIC/2	16 (0.50)^S^	32 (0.50)^S^	64 (1)^I^	64 (˂0.25)^S^	4 (0.50)^S^	16 (0.25)^S^	32 (0.25)^S^	4 (0.25)^S^	128(1)^I^	7/9 (77.78 %)
	MIC/5	32 (1)^I^	64 (1)^I^	64 (1)^I^	128 (˂0.50)^S^	1 (0.13)^S^	32 (0.50)^S^	64 (0.50)^S^	16 (1)^I^	128(1)^I^	4/9 (36.36 %)
	*Polyscias fulva*										
CIP	0	4	64	4	64	4	128	32	16	32	
	MIC/2	4 (1)^I^	64 (1)^I^	2 (0.50)^S^	64 (1)^I^	4 (1)^I^	128 (1)^I^	2 (0.06)^S^	8 (0.50)^S^	32(1)^I^	3/9 (27.27 %)
	MIC/5	4 (1)^I^	64 (1)^I^	2 (0.50)^S^	64 (1)^I^	4 (1)^I^	128 (1)^I^	4 (0.13)^S^	16 (1)^I^	32(1)^I^	2/9 (22.22 %)
TET	0	8	64	64	32	2	64	32	64	16	
	MIC/2	2 (0.25)^S^	32 (0.50)^S^	32 (0.50)^S^	4 (0.13)^S^	<0.50 (<0.25)^S^	32 (0.50)^S^	8 (0.25)^S^	2 (0.03)^S^	8(0.50)^S^	9/9 (100 %)
	MIC/5	4 (0.50)^S^	64 (1)^I^	32 (0.50)^S^	16 (0.50)^S^	2 (1)^I^	32 (0.50)^S^	32 (1)^I^	32 (0.50)^S^	8(0.50)^S^	6/9 (66.67 %)
KAN	0	-	16	128	4	16	16	64	4	128	
	MIC/2	8(˂0.06)^S^	8 (0.50)^S^	64 (0.50)^S^	2 (0.50)^S^	2 (0.13)^S^	8 (0.50)^S^	32 (0.50)^S^	<1 (<0.25)^S^	64(0.50)^S^	9/9 (100 %)
	MIC/5	64 (˂0.50)^S^	16 (1)^I^	128 (1)^I^	4 (1)^I^	16 (1)^I^	8 (0.50)^S^	32 (0.50)^S^	4 (1)^I^	64(0.50)^S^	4/9 (36.36 %)
AMP	0	-	-	-	-	-	-	-	-	-	
	MIC/2	128 (˂0.50)^S^	-(≥1)	256 (˂1)^S^	-(≥1)	256 (˂1)^S^	256 (˂1)^S^	-(≥1)	256 (˂1)^S^	-(>1)	5/9 (55.55 %)
	MIC/5	256 (˂1)^S^	-(≥1)	-(≥1)	-(≥1)	-(≥1)	-(˃1)	-(≥1)	-(≥1)	-(>1)	1/9 (11.11 %)
CHL	0	32	64	64	-	8	64	128	16	128	
	MIC/2	16 (0.50)^S^	64 (1)^I^	64 (1)^I^	128 (˂0.50)^S^	4 (0.50)^S^	8 (0.13)^S^	64 (0.50)^S^	2 (0.13)^S^	64(0.50)^S^	7/9 (77.78 %)
	MIC/5	32 (1)^I^	64 (1)^I^	64 (1)^I^	256 (˂1)^S^	4 (0.50)^S^	16 (0.25)^S^	64 (0.50)^S^	16 (1)^I^	64(0.50)^S^	5/9 (55.55 %)

^a^Antibotics [*TET* tetracycline, *CIP* ciprofloxacin, *KAN* kanamycin, *CHL* chloramphenicol, *AMP* ampicillin]. ^b^Bacterial strains: *Escherichia coli* [AG102, AG100Atet], *Pseudomonas aeruginosa* [PA01, PA124], *Enterobacter aerogenes* [CM64, EA27], *Enterobacter cloacae* [BM67], *Klebsiella pneumoniae* [KP55], *Providencia stuartii* [NAE16]. ^c^ *PBSS* percentage of bacteria strain on which synergism has been observed, *NA* not applicable

(): fold increase in MIC values of the antibiotics after association with plants extract, *S* synergy, *I* indifference, *na* not applicable, *FIC* fractional inhibitory concentration, (−): >256 μg/mL, 0: no extract (only antibiotic tested)

## Discussion

Phytochemicals are routinely classified as antimicrobials on the basis of susceptibility tests that produce MICs in the range of 100 to 1000 μg/mL [27]. Moreover, for crude extracts, the antimicrobial activity is considered to be significant if MIC values are below 100 μg/mL and moderate when 100< MIC <625 μg/mL [28, 29]. Therefore, the activity recorded with *B. acuta* bark extract against the 26 tested bacterial strains can be considered as very important. If we consider the alternative criteria described by Fabry et al*.* [30], where extracts having MIC values less than 8000 μg/mL have noteworthy antimicrobial activity, the overall activity recorded with the leaves and fruit extracts of *B. acuta, P. fulva* and *N. laevis* leaves extracts can also be considered promising*.* A keen look of the results of MIC and MBC determinations (Table 3, Additional file 1: Tables S1 and S2) indicates that MBC/MIC ratios were mostly above four, suggesting that studied extracts, including the most active ones, generally displayed bacteriostatic effects (MBC/MIC > 4) [31–33]*.* Various classes of phytochemicals (Table 2) were previously detected in the extracts of the four tested plants [10] and this may explain their antibacterial activity.

The results obtained in this study, and mostly those obtained with the bark of *B. acuta* are very important when taking in consideration the fact that most of the bacterial strains used were MDR phenotypes expressing active efflux pumps [7–9, 34, 35]. In fact, the activity of antibiotics against the studied MDR bacteria was previously found to increase in the presence of phenylalanine arginine β-naphthylamide (PAßN), a potent inhibitor of RND efflux systems, particularly AcrAB–TolC (of Enterobaceriaceae) and MexAB–OprM (of *Pseudomonas* species) [7–9, 34, 35]. In the present study, we demonstrated that beneficial effects when combining four of the tested plant extracts [namely those from *B. acuta* (leaves and bark), *N. leavis* (leaves) and *P. fulva* (leaves)] with the first line antibiotics could be achieved. High percentages of synergistic effects (100 %) obtained with *B. acuta* bark extract and TET as well as *P. fulva* leaves extract in combination with TET and KAN, clearly suggest that such associations could improve the fight against MDR bacterial infections. This also suggests that some of the constituents of the corresponding plants can act as efflux pump inhibitors, as more than 70 % synergistic cases were observed with many combinations [26].

The antimicrobial potential of the genus *Beilschmiedia* has previously been documented. Chouna et al. [36] demonstrated that compounds such as beilschmiedic acid C isolated from *B. anacardioides* were significantly active against *Bacillus subtilis*, *Micrococcus luteus* and *Streptococcus faecalis. Beilschmiedia cinnamomea* was previously reported to have significant to moderate activities (64–1024 μg/mL) against the MDRGN tested in this work [7]. *Beilschmedia obscura* was also found to show a good and large spectrum of antibacterial activity against MDRGN [37]. Some compounds previously isolated from the genus *Beilschmiedia* and belonging to alkaloids, phenols, saponines, sterols and triterpenoids [36, 38] were shown to possess antimicrobial activities [7]. The genus *Beilschmiedia* is also known traditionally to possess antimicrobial activities [7]. *Beilschmedia acuta* tested in this study is also used in Cameroon to treat gastrointestinal infections [10]. The obtained data highlight the importance of this plant in the control of microbial infections and mostly those involving MDR phenotypes. The antimicrobial activities of extracts and compounds from *Newbouldia laevis* towards sensitive bacteria and fungi were also reported [39, 40], and the present study provides additional data on the potential of this plant to fight MDR bacteria. Also, the antimicrobial activity of essential oil from *Clausena anisata* was reported against *Staphylococcus aureus, Streptococcus pneumoniae, Enterococcus species*, *Salmonella typhimurium* and *Pseudomonas aeruginosa* [41, 42]. The present report provides more evidence of the antimicrobial potential of this plant.

## Conclusion

The results of this study are very interesting, in regards to the medical importance of the studied microorganisms. These data provided evidence that crude extracts from the studied plants and mostly that from the bark of *Beilschmedia acuta* are potential sources of antimicrobial drugs to fight MDR bacterial infections. The purification of this plant will be carried out to isolate its active constituents. The cytotoxicity assays on normal cell lines constitute the limitation of the present work and will further be performed to ensure the safety of the tested extracts.

## Additional file

Additional file 1: Table S1. MICs up to 1024 μg/mL of the crude extracts and ciprofloxacin on the panel of tested bacteria. **Table S2.** MBCs up to 1024 μg/mL of the crude extracts and ciprofloxacin on the panel of tested bacteria. (DOCX 29 kb)
