# Supplementary material for: In vitro antibacterial and antibiotic-potentiation activities of the methanol extracts from Beilschmiedia acuta, Clausena anisata, Newbouldia laevis and Polyscias fulva against multidrug-resistant Gram-negative bacteria
Source: BMC Complement Altern Med. 2015 Nov 22;15:412. doi: 10.1186/s12906-015-0944-5 (PMC4655084; doi:10.1186/s12906-015-0944-5)
Supplement: Additional file 1: Table S1. — MICs up to 1024 μg/mL of the crude extracts and ciprofloxacin on the panel of tested bacteria. Table S2. MBCs up to 1024 μg/mL of the crude extracts and ciprofloxacin on the panel of tested bacteria. (DOCX 29 kb) [file 12906_2015_944_MOESM1_ESM.docx]

***In Vitro* Antibacterial and Antibiotic-potentiation Activities of the Methanol Extracts from *Beilschmiedia acuta, Clausena anisata*, *Newbouldia laevis* and *Polyscias fulva* against Multidrug-Resistant Gram-Negative Bacteria**

Simplice B. Tankeo^1^, Pierre Tane^2^ and Victor Kuete^1*^

*^1^Department of Biochemistry, Faculty of Science, University of Dschang, Cameroon;*

*^2^Laboratory of Natural Products Chemistry, Department of Chemistry, Faculty of Science, University of Dschang, Cameroon;*

**Corresponding authors:**

**Tel : (237) 77 35 59 27 ; Fax: (237) 22 22 60 18. P.O. Box 67 Dschang, Cameroon; E-mail:* [*kuetevictor@yahoo.fr*](mailto:kuetevictor@yahoo.fr) *(Prof. Dr. Victor Kuete)*

***Authors e-mails:***

*Simplice B. Tankeo: presidentthankeo@yahoo.fr,*

*Pierre Tane: ptane@yahoo.fr,*

*Victor Kuete:* [*Kuetevictor@yahoo.fr*](mailto:Kuetevictor@yahoo.fr)

**Table S1.** MICs up to 1024 µg/mL of the crude extracts and ciprofloxacin on the panel of tested bacteria

| Bacterial strains | Studied samples and MIC (µg/mL) | | | | | | | | | | |
| --- | --- | --- | --- | --- | --- | --- | --- | --- | --- | --- | --- |
|  | *Beilschmedia acuta* | | | *Clausena anisata* | | *Newbouldia laevis* | | *Polyscias fulva* | |  | Reference drug |
|  | L | B | F | L | R | L | B | L | B | R | CIP |
| *Escherichia coli* |  |  |  |  |  |  |  |  |  |  |  |
| ATCC 10536 | 1024 | 64 | 1024 | 256 | 256 | 128 | 1024 | 1024 | - | 1024 | 1 |
| AG 100A | 512 | 128 | 1024 | 512 | 1024 | 256 | 1024 | 512 | - | - | <0.5 |
| AG 100 | 1024 | 16 | 1024 | - | 1024 | 1024 | 1024 | 512 | 1024 | - | 16 |
| AG 100A_Tet_ | 1024 | 256 | 1024 | - | 1024 | 1024 | - | 1024 | - | - | 64 |
| AG 102 | 512 | 64 | 512 | 512 | 1024 | 512 | 1024 | 512 | 1024 | 512 | 4 |
| MC 4100 | 256 | 128 | 256 | 256 | 256 | 128 | 1024 | 256 | 512 | 256 | 16 |
| W 3110 | 256 | <8 | 256 | 256 | 256 | 128 | 1024 | 128 | 1024 | 128 | 32 |
| *Enterobacter aerogenes* |  |  |  |  |  |  |  |  |  |  |  |
| ATCC 13048 | 512 | 16 | 1024 | 1024 | 1024 | 1024 | - | 256 | 1024 | 512 | 32 |
| CM 64 | 1024 | 16 | 1024 | - | 1024 | 1024 | 1024 | 1024 | - | - | 64 |
| EA3 | 1024 | 64 | 1024 | - | - | 1024 | 1024 | 1024 | - | - | 16 |
| EA27 | 512 | 64 | 1024 | - | 512 | 128 | - | 256 | - | - | 4 |
| EA 294 | 1024 | 64 | 512 | 512 | 256 | 256 | 1024 | 512 | 1024 | 512 | 2 |
| EA 289 | 512 | 256 | 256 | - | 1024 | 1024 | 1024 | 512 | - | 1024 | 128 |
| EA 298 | 1024 | 256 | 256 | - | 1024 | 1024 | 1024 | 1024 | - | 1024 | 16 |
| *Klebsiella pneumoniae* |  |  |  |  |  |  |  |  |  |  |  |
| ATCC11296 | 128 | <8 | 256 | 256 | 256 | 128 | 512 | 128 | 256 | 128 | <0.5 |
| K2 | 1024 | 256 | 1024 | - | - | 1024 | 1024 | - | - | - | 16 |
| KP55 | 512 | 32 | 1024 | - | - | 1024 | - | 1024 | - | - | 4 |
| KP63 | 128 | 64 | 128 | 256 | 512 | 256 | 1024 | 128 | 512 | 128 | 4 |
| *Providencia stuartii* |  |  |  |  |  |  |  |  |  |  |  |
| ATCC29916 | 512 | 128 | 512 | - | 1024 | 1024 | 1024 | 1024 | - | - | 32 |
| PS2636 | 256 | 64 | 256 | 256 | 128 | 128 | 512 | 256 | 1024 | 512 | 64 |
| PS299645 | 1024 | 256 | 1024 | - | 512 | 1024 | 1024 | 1024 | - | - | 32 |
| NAE16 | 512 | 32 | 512 | - | 1024 | 1024 | - | 1024 | - | - | 128 |
| *Enterobacter cloacae* |  |  |  |  |  |  |  |  |  |  |  |
| ECCI69 | 1024 | 256 | 1024 | - | - | 1024 | 1024 | 512 | 1024 | 1024 | 256 |
| BM67 | 1024 | 256 | 1024 | ­­- | ­- | - | - | 1024 | - | - | 32 |
| *Pseudomonas aeruginosa* |  |  |  |  |  |  |  |  |  |  |  |
| PA01 | 1024 | 64 | 256 | 1024 | 512 | 1024 | 1024 | 512 | - | 1024 | 16 |
| PA124 | 512 | 32 | 1024 | - | - | - | - | - | - | - | 32 |

The tested extracts were obtianed from the leaves (L), bark (B), roots (R)or fruits (F); CIP : ciprofloxacin]; (-): MIC>1024 µg/mL

**Table S2.** MBCs (µg/mL) of the crude extracts and ciprofloxacin on the panel of tested bacteria

| Bacterial strains | Studied samples and MBC (µg/mL) | | | | | | | | | | |
| --- | --- | --- | --- | --- | --- | --- | --- | --- | --- | --- | --- |
|  | *Beilschmedia acuta* | | | *Clausena anisata* | | *Newbouldia laevis* | | *Polyscias fulva* | | | Reference drug |
|  | L | B | F | L | R | L | B | L | B | R | CIP |
| *Escherichia coli* |  |  |  |  |  |  |  |  |  |  |  |
| ATCC 10536 | - | 1024 | - | - | - | - | - | - | - | - | 4 |
| AG 100A | - | 1024 | - | - | - | - | - | - | - | - | 1 |
| AG 100 | - | 128 | - | - | - | - | - | - | - | - | 64 |
| AG 100A_Tet_ | - | **-** | - | - | - | - | - | - | - | - | 128 |
| AG 102 | - | 256 | - | - | - | - | - | - | - | - | 16 |
| MC 4100 | - | 128 | - | - | - | 1024 | - | - | - | - | 64 |
| W 3110 | 1024 | 256 | 1024 | - | - | - | - | 1024 | - | - | 64 |
| *Enterobacter aerogenes* |  |  |  |  |  |  |  |  |  |  |  |
| ATCC 13048 | 1024 | 512 | - | - | - | - | - | 1024 | - | - | 256 |
| CM 64 | - | 512 | - | - | - | - | - | - | - | - | 256 |
| EA3 | - | 512 | - | - | - | - | - | - | - | - | 256 |
| EA27 | 1024 | 128 | - | - | - | - | - | - | - | - | 16 |
| EA 294 | - | 512 | - | - | 1024 | - | - | - | - | - | 256 |
| EA 289 | - | 1024 | 1024 | - | - | - | - | - | - | - | 128 |
| EA 298 | - | 1024 | 1024 | - | - | - | - | - | - | - | 16 |
| *Klebsiella pneumoniae* |  |  |  |  |  |  |  |  |  |  |  |
| ATCC11296 | - | 256 | - | - | ­- | 1024 | - | - | - | - | 128 |
| K2 | - | - | - | - | - | - | - | - | - | - | 16 |
| KP55 | - | 512 | - | - | - | - | - | - | - | - | 16 |
| KP63 | 1024 | 256 | 1024 | - | - | - | - | 512 | 1024 | 512 | 16 |
| *Providencia stuartii* |  |  |  |  |  |  |  |  |  |  |  |
| ATCC29916 | 1024 | 256 | - | - | - | - | - | - | - | - | 256 |
| PS2636 | - | 512 | - | - | 1024 | - | - | - | - | - | 256 |
| PS299645 | - | 1024 | - | - | - | - | - | - | - | - | 128 |
| NAE16 | - | 512 | - | - | - | - | - | - | - | - | 256 |
| *Enterobacter cloacae* |  |  |  |  |  |  |  |  |  |  |  |
| ECCI69 | - | 1024 | - | - | - | - | - | - | - | - | >256 |
| BM67 | - | - | - | - | - | - | - | - | - | - | 256 |
| *Pseudomonas aeruginosa* |  |  |  |  |  |  |  |  |  |  |  |
| PA01 | - | 1024 | - | - | - | - | - | - | - | - | 64 |
| PA124 | - | 1024 | - | - | - | - | - | - | - | - | 256 |

The tested extracts were obtianed from the leaves (L), bark (B), roots (R)or fruits (F); CIP : ciprofloxacin]; (-): MBC>1024 µg/mL
